# Peripheral Calcifying Epithelial Odontogenic Tumor Masquerading as Pyogenic Granuloma of Oral Cavity

**DOI:** 10.1002/ccr3.71137

**Published:** 2025-10-05

**Authors:** Saravanan Sampoornam Pape Reddy, Delfin Lovelina Francis, Deepak Pandiar, Ruchi Harish, Manish Rathi, Ravikiran Narayana, Balaji Manohar, Shaili Pradhan

**Affiliations:** ^1^ 21 Corps Dental Unit Army Dental Corps New Delhi India; ^2^ Saveetha Dental College & Hospitals Saveetha University, SIMATS Chennai India; ^3^ Department of Periodontology Darshan Dental College and Hospitals Udaipur Rajasthan India; ^4^ Department of Periodontology, Geetanjali Dental and Research Institute Geetanjali University Udaipur India; ^5^ Department of Periodontology & Oral Implantology Kathmandu Medical College Public Limited Kathmandu Nepal

**Keywords:** calcifying epithelial odontogenic tumor, case report, gingiva, pyogenic granuloma

## Abstract

We presented a case of PCOT that initially displayed clinical and histological features similar to pyogenic granuloma. Excisional biopsy and histological examination were performed to determine the definitive diagnosis. Therefore, it is argued that the clinical and incisional biopsy findings represent only alterations in the submitted tissue, which may be deceptive, and the final diagnosis may differ following surgical excision.

## Graphical abstract

A 48‐year‐old female presented with a progressive gingival swelling in the right maxillary canine region, initially misdiagnosed as pyogenic granuloma due to overlapping features with peripheral ossifying fibroma, peripheral giant cell granuloma, odontogenic tumors, and squamous cell carcinoma. Definitive diagnosis was established only after complete excision and histopathological analysis, which revealed polyhedral epithelial cells arranged in sheets and islands, amyloid‐like deposits, and characteristic Liesegang ring calcifications, confirming a peripheral calcifying epithelial odontogenic tumor (PCOT). PCOT is a rare, benign, and slow‐growing odontogenic neoplasm with an excellent prognosis following surgical excision, though rare clear cell variants may present a higher recurrence risk. This case highlights the importance of integrating clinical, radiographic, and histopathological findings, while emphasizing that excisional biopsy and special stains such as Congo Red and Thioflavin S are crucial for confirming the diagnosis and preventing recurrence.

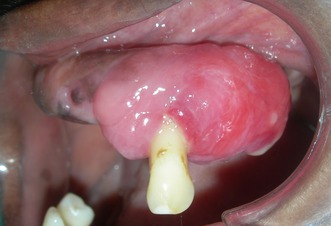

**Summary**

- It is imperative to keep in mind that the etiopathogenesis of peripheral calcifying epithelial odontogenic tumor (PCOT) involves a combination of genetic, environmental, and molecular factors.
- PCOT is typically treated with surgical excision and followed up for recurrence.
- Clinicians should maintain a high index of suspicion in similar cases to prevent complications.

Peripheral calcifying epithelial odontogenic tumor (PCOT), also known as Pindborg tumor, is an extremely rare and benign neoplasm originating from odontogenic epithelium.

A 48‐year‐old female presented with the chief complaint of a growth in the mouth that progressively grew larger over the course of a year. The medical history was noncontributory. Dental history revealed multiple dental extractions due to mobility of teeth. A 35 × 27 × 15 mm sized diffuse, sessile swelling encircling the right maxillary canine (MRC) was present that was soft and edematous on the mesial side, erythematous on the distal half, and pustulous at the distal end of the lesion (Figure 1). A provisional diagnosis of pyogenic granuloma was established and an incisional biopsy was performed along with extraction of MRC.

**FIGURE 1:**
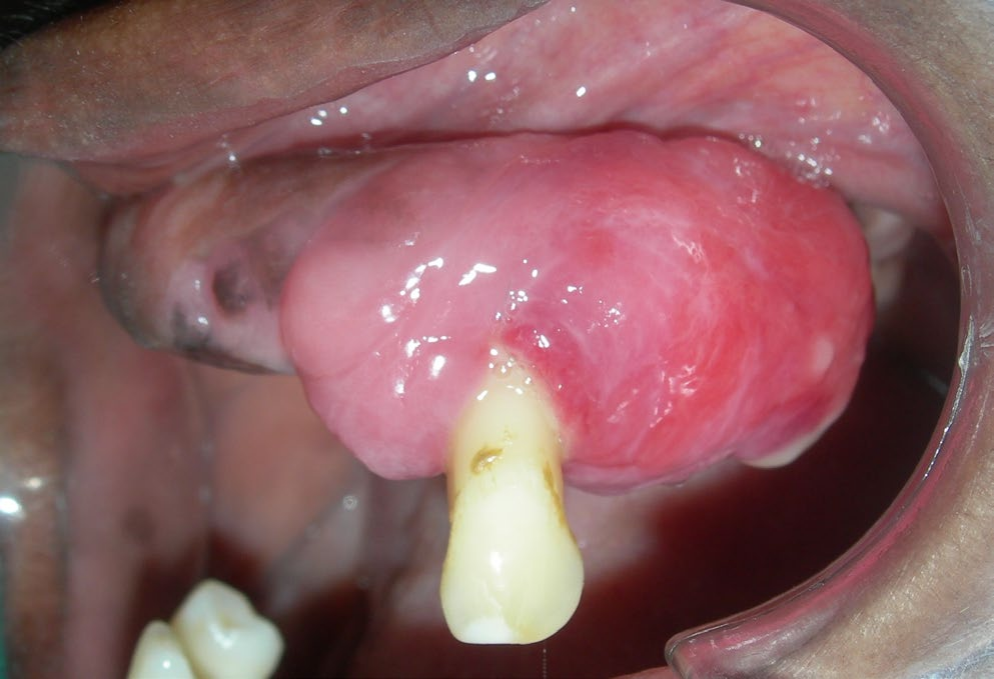
Clinical presentation.

Differential diagnoses included peripheral ossifying fibroma, pyogenic granuloma, peripheral giant cell granuloma (PGCG), peripheral ameloblastoma, peripheral odontogenic fibroma (POF), squamous cell carcinoma (SCC), and calcifying odontogenic cyst (COC, Gorlin Cyst).

Accurate diagnosis is crucial for determining appropriate management and preventing recurrence. While PCOT is a peripheral lesion and usually does not involve the bone, radiographic imaging is helpful to detect if there is any underlying bony involvement, erosion, or calcifications. Histopathological examination is critical for the definitive diagnosis of PCOT. If an incisional biopsy is inconclusive or if the lesion does not regress following initial treatment, an excisional biopsy may be warranted.

PCOT is a rare, benign lesion that requires a combination of clinical, radiographic, and histopathological evaluations for accurate diagnosis. Histopathology remains the gold standard for confirming the diagnosis, especially with the presence of characteristic amyloid‐like deposits and 'Liesegang ring' calcifications. One month after extraction and incisional biopsy, there were no notable alterations to the swelling other than a firm and fibrous appearance. The lesion was then completely excised, and HPE was performed, which revealed polyhedral epithelial cells with ‘tear drop’ shaped rete pegs, subepithelial lymphocytic infiltration, extracellular eosinophilic amyloid‐like deposits, overall reduction in connective tissue volume which was scant and numerous ‘ring‐like’ calcifications with ‘cribriform appearance’ typical of ‘Liesegang Rings’ (Figures 2 and 3). The definitive final diagnosis was determined to be PCOT based on these findings. Usage of special stains such as Congo Red or Thioflavin S is usually recommended for confirmation of the presence of amyloid deposits.

**FIGURE 2:**
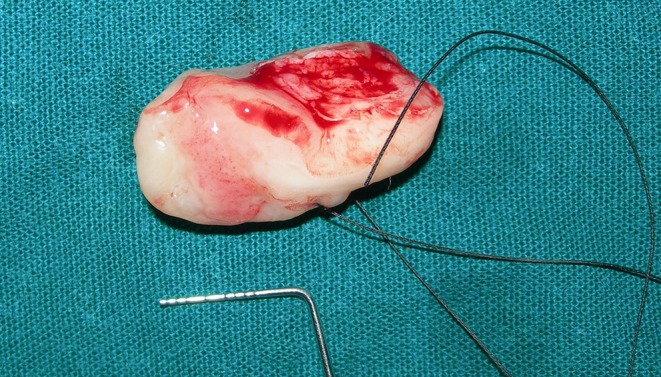
Excised lesion.

**FIGURE 3:**
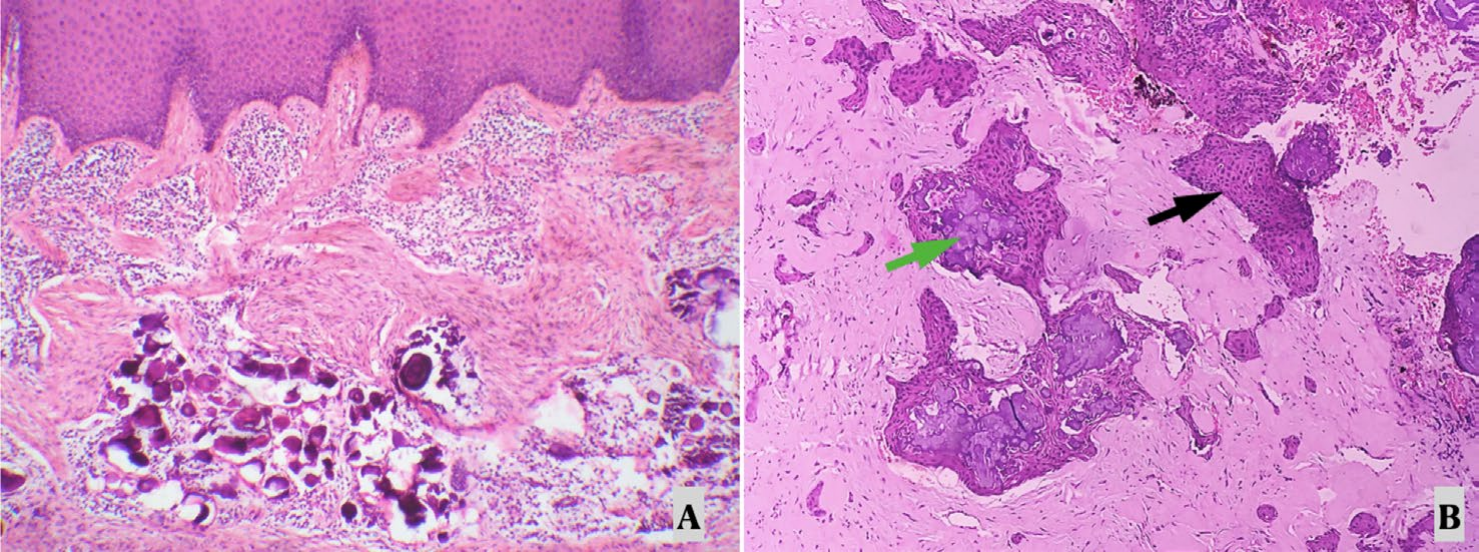
Definitive histopathological examination. (A) Low power 20× and (B) high power 40×, showing sheets of polyhedral epithelial cells (black arrow) and the basophilic to amphophilic acellular amyloid deposits (green arrow).

PCOT is a rare tumor and its incidence is uncommon compared to other odontogenic tumors, with a high prevalence in the posterior mandible as opposed to the present case, which was in the anterior maxilla [1]. It is benign and slowly growing, with ‘calcifications’ being one of the pathognomonic features under HPE [2]. Although the primary treatment for PCOT is surgical excision including a margin of healthy tissue to ensure complete removal, the prognosis is excellent in the majority of cases with an extremely low recurrence rate with the exception of clear cell variants, which are extremely uncommon (5%–6% of reported cases of PCOT) [3]. Routine dental health screenings and follow‐up sessions are critical in these cases. The patient was then observed for the next 1 year, during which time an interim prosthesis was provided and a final prosthesis was planned.

## Author Contributions

**Saravanan Sampoornam Pape Reddy:** conceptualization, data curation, formal analysis. **Delfin Lovelina Francis:** formal analysis, investigation, methodology. **Deepak Pandiar:** data curation, formal analysis, investigation. **Ruchi Harish:** resources, validation. **Manish Rathi:** investigation, resources. **Ravikiran Narayana:** writing – original draft. **Balaji Manohar:** writing – review and editing. **Shaili Pradhan:** supervision, validation, visualization.

## Ethics Statement

The authors have nothing to report.

## Consent

A written informed consent form was obtained from the patient reported in this case image.

## Conflicts of Interest

The authors declare no conflicts of interest.

## Data Availability

There is no data generated from this case report.
